# Polyphenols as Plant-Based Nutraceuticals: Health Effects, Encapsulation, Nano-Delivery, and Application

**DOI:** 10.3390/foods11152189

**Published:** 2022-07-23

**Authors:** Zhiheng Zhang, Xiaojing Li, Shangyuan Sang, David Julian McClements, Long Chen, Jie Long, Aiquan Jiao, Zhengyu Jin, Chao Qiu

**Affiliations:** 1 State Key Laboratory of Food Science and Technology, School of Food Science and Technology, Collaborative Innovation Center of Food Safety and Quality Control in Jiangsu Province, Jiangnan University, Wuxi 214122, China; 6200112177@stu.jiangnan.edu.cn (Z.Z.); longchen@jiangnan.edu.cn (L.C.); jielong@jiangnan.edu.cn (J.L.); aqjiao@jiangnan.edu.cn (A.J.); fpcenter@jiangnan.edu.cn (Z.J.); 2 College of Light Industry and Food Engineering, Nanjing Forestry University, Nanjing 210037, China; lxj0810jy@163.com; 3 College of Food and Pharmaceutical Sciences, Ningbo University, 169 Qixing South Road, Ningbo 315832, China; shangyuan.sang@foxmail.com; 4 Department of Food Science, University of Massachusetts, Amherst, MA 01060, USA; mcclemen@umass.edu

**Keywords:** polyphenol compounds, functional food, nano-delivery, bioavailability, health

## Abstract

Plant polyphenols have attracted considerable attention because of their key roles in preventing many diseases, including high blood sugar, high cholesterol, and cancer. A variety of functional foods have been designed and developed with plant polyphenols as the main active ingredients. Polyphenols mainly come from vegetables and fruits and can generally be divided according to their structure into flavonoids, astragalus, phenolic acids, and lignans. Polyphenols are a group of plant-derived functional food ingredients with different molecular structures and various biological activities including antioxidant, anti-inflammatory, and anticancer properties. However, many polyphenolic compounds have low oral bioavailability, which limits the application of polyphenols in nutraceuticals. Fortunately, green bio-based nanocarriers are well suited for encapsulating, protecting, and delivering polyphenols, thereby improving their bioavailability. In this paper, the health benefits of plant polyphenols in the prevention of various diseases are summarized, with a review of the research progress into bio-based nanocarriers for the improvement of the oral bioavailability of polyphenols. Polyphenols have great potential for application as key formulations in health and nutrition products. In the future, the development of food-grade delivery carriers for the encapsulation and delivery of polyphenolic compounds could well solve the limitations of poor water solubility and low bioavailability of polyphenols for practical applications.

## 1. Introduction

**Figure 1:**
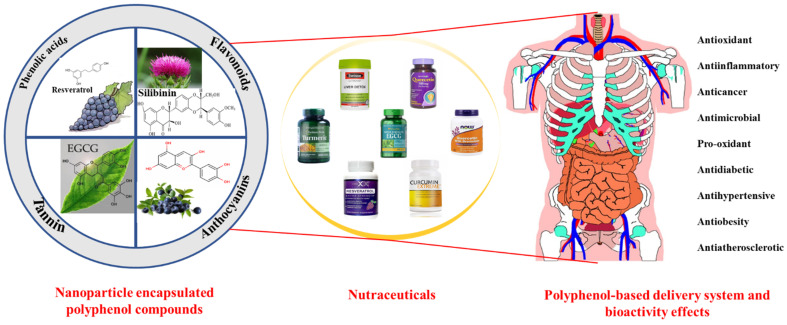
Beneficial effects of polyphenol-based delivery systems and their potential application in nutraceutical formulations.

Plant-derived functional foods have gradually become an area of increasing research interest because of their health benefits to the human body, and plant polyphenols have gained widespread attention as one of the most abundant and widely distributed chemical components. Plant polyphenols naturally occur in vegetables, fruits, cereals, tea, coffee, and other plants. These compounds take phenolic rings as the basic monomer and have a variety of complex structures, which are generally divided into phenolic acids, flavonoids, anthocyanins, and tannins (Figure 1) [1,2]. Some foods contain a variety of bioactive compounds that can cause a variety of biological effects when interacting with the body; these are known as bioactive substances. Plant-based foods contain bioactive substances, including polyphenols [3,4]. Some can be directly isolated and extracted from natural foods, while others can be obtained by reprocessing natural ingredients. Polyphenols mainly depend on biological use to express their biological properties, and their absorption speed and the limits of their activity in the gut depend on their chemical structure [5,6]. Polyphenols are considered good preventive agents for chronic and degenerative diseases, due to their extensive and special chemical structure that endows them with many biological functions. Food polyphenols are common antioxidants in food that can effectively prevent atherosclerosis and prevent aging [7]. Some studies have also confirmed the beneficial effects of polyphenols in the prevention of cardiovascular diseases, neurological diseases, liver diseases, diabetes, and cancer [8].

Polyphenols are widely found in plant-based foods, and many polyphenols have anti-inflammatory, antioxidant, and anticancer properties [9,10]. Long-term consumption of foods rich in plant polyphenols has been shown to improve conditions as diverse as diabetes, cancer, osteoporosis, neurodegenerative diseases, and cardiovascular disease [11]. Other studies have found that polyphenols have a variety of beneficial effects on intestinal health and are potential prebiotics, able to play a variety of probiotic roles including reducing inflammation and preventing cancer by regulating the intestinal flora [12,13]. Therefore, they are often used as additives in functional dietary foods to achieve beneficial effects through food intake. The market demand for polyphenols is increasing rapidly with social and economic development and the improvement of people’s health awareness. However, many polyphenolic compounds have low oral bioavailability, which limits the application of polyphenols in nutraceuticals.

Fortunately, green bio-based nanocarriers are well suited for encapsulating, protecting, and delivering polyphenols, thus enhancing their bioavailability (Figure 1) [1]. Bio-based polymers (e.g., proteins and polysaccharides) with good biocompatibility, biodegradability, resource sustainability, and good nutritional value are excellent delivery vehicles to address the limitations of low oral utilization of polyphenols when applied as nutritional medicines. Protein is a multifunctional bio-based polymer with rich nutritional value and various functional properties, including emulsification, amphiphilicity, gelation, and foaming properties. Its unique chemical structure and functionality allow it to be engineered into nanoparticles, nanogels, nanoemulsions, nanofilms, and nanofibers that can deliver both hydrophilic and hydrophobic polyphenolic compounds [14]. The unique structure and physiological activity of polysaccharides, as bio-based polymers with a wide range of origins, allow them to be used as raw materials for the preparation of various types of nanocarriers and for the delivery of polyphenolic compounds [1]. In addition, the good biocompatibility and biodegradability of lipid-based nanocarriers make them one of the key technologies for the delivery of polyphenols in the field of food and nutrition. Lipid-based delivery vectors (including liposomes, nano-emulsions, and solid lipid nanoparticles) are good ways to protect fat-soluble polyphenols in the gastrointestinal tract and improve their bioavailability [1]. Some commercialized functionalized polyphenol products have been introduced onto the market, which undoubtedly provides a foundation for the further exploration and development of new polyphenol health nutrition products [15,16].

In this article, we provide an overview of the classification of polyphenolic compounds and an exhaustive summary of the health benefits of polyphenols for human health improvement. We also propose improvements to address the limitations of polyphenolic compounds in their application as health food formulations: namely, the design and preparation of food-grade bio-based nanocarriers to encapsulate, protect, and deliver polyphenolic compounds, thereby improving their bioavailability. Finally, we look forward to the prospective application of polyphenol food-grade bio-based nanocomplexes in nutraceuticals, with the aim of providing valuable insights into the development and utilization of nutraceuticals through the combination of nano-delivery methods and the health benefits of polyphenols.

## 2. Classification, Properties, and Beneficial Effects of Plant Polyphenols

### 2.1. Classification and Properties

Plant polyphenols are widely found in various foods and have attracted much research attention because of their antioxidant, anti-inflammatory, and anticancer biological activities. Polyphenols generally refer to chemicals with a benzene ring structure and two or more phenolic hydroxyl groups, which are generally classified into flavonoids and phenolic acids according to their structural characteristics [1].

**Figure 2:**
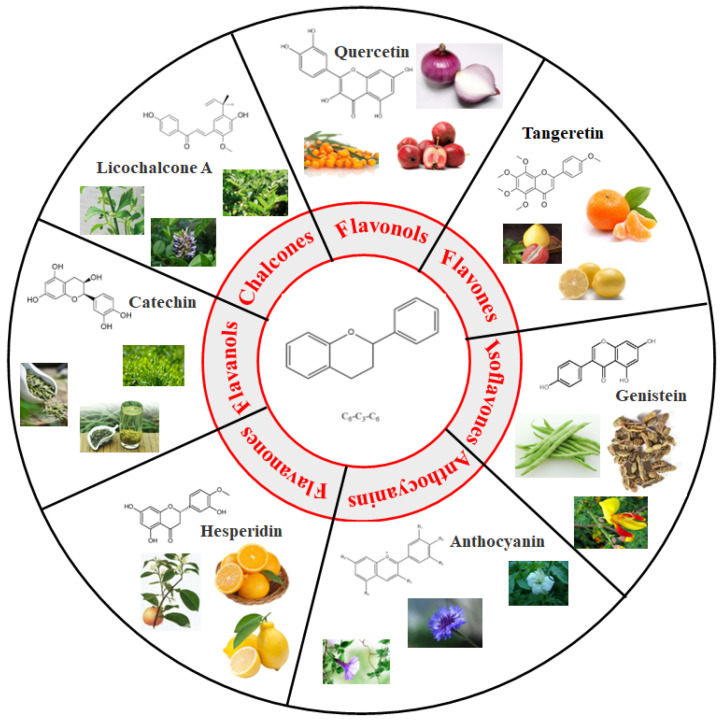
Classification of flavonoids and examples.

Generally, flavonoids exist mainly in the form of glycosides in the vesicles of plant cells. Structurally, flavonoids have three cyclic structures of C6–C3–C6 as their basic skeleton. According to the differences in chemical structures, flavonoids are further subdivided into flavonols, flavones, isoflavones, anthocyanins, flavanones, flavanols, and chalcones (Figure 2). Most plants contain flavonoids that play an important role in plant growth, development, flowering, fruiting, antibacterial activity, and disease prevention. Most of these flavonoids have anti-inflammatory, antibacterial, antioxidant, and anticancer physiological activities, and can have beneficial effects on the human body (Table 1).

Phenolic acids are widely found in fruits, vegetables, and beverages. Low molecular weight phenolic acids exhibit water-soluble properties during further processing, as well as during human digestion, and when phenolic acids undergo condensation reactions with glucose and quinic acid, they assume a water-insoluble state [17]. Phenolic acids are generally classified into monohydroxybenzoic acid, dihydroxybenzoic acid, and trihydroxybenzoic acid, according to their hydroxyl content. Phenolic acids are widely used in various functional foods because of their good antioxidant, anti-inflammatory, antiallergic, and anticancer properties, as well as cardioprotective and other health-functional activities [18].

### 2.2. Beneficial Effects

Plant polyphenols have a variety of beneficial effects on human health. Their excellent biological activities such as antioxidant and antibacterial effects, as well as their natural availability and biocompatibility, make it possible for them to be added to foods and endowed with unique functional properties to exert their beneficial effects on human health. Some of the beneficial effects of plant polyphenols on human health are presented in Table 2, showing the promising application of polyphenols in functional food formulations. The potential role of functional foods containing polyphenolic compounds is extremely important in the prevention of many chronic diseases including diabetes, hypertension, and cancer.

#### 2.2.1. Antioxidant Effect

Plant polyphenolic compounds generally have good antioxidant activity due to their specific structural characteristics. Fruits and vegetables, as well as some cereals, are rich in polyphenolic compounds, which play an important role in human health. Polyphenols as antioxidants can prevent several diseases by scavenging free radicals and thus protecting DNA from oxidative damage. Jakubczyk et al. [38] conducted a search of PubMed and Embase for randomized clinical trials of >20 patients treated with curcumin supplements from the start of the database until 27 September 2019. A total of 308 participants were enrolled in the study on the antioxidant potential of curcumin. A total of 40% of the respondents were men. The mean age of the participants was 27.60 ± 3.79 years. The average duration of supplementation was 67 days, and the average dose of curcumin administered was 645 mg/24 h. The results showed that curcumin had good antioxidant capacity and tended to reduce malondialdehyde concentrations. In a study by Grzesik et al. [39], the antioxidant capacities of catechins, glutathione, and ascorbic acid were compared. It was found that catechins were the most effective for scavenging ABTS radicals, had the highest reduction equivalents for trivalent iron ions, and were effective in protecting against the oxidation of dihydrorhodamine. The excellent antioxidant properties of polyphenolic compounds such as catechins make them ideal candidates for antioxidant prophylaxis and therapy.

#### 2.2.2. Anti-Inflammatory Effect

Plant polyphenols have good inhibitory and killing effects on some inflammatory cells, either by affecting cytokines and their receptors or by influencing their secretion processes. It was reported that hydrogels containing rutin exhibited good anti-inflammatory activity comparable to that of standard drugs, as demonstrated in the study of Singhai et al. [46]. Hesperidin was evaluated by Ding et al. [47]. Hesperidin was tested for anti-inflammatory activity using RAW264.7 cells and a CCl4-induced acute liver injury model, and it was found that hesperidin was effective in reducing nitric oxide (NO), interleukin (IL-6), and tumor necrosis factor (TNF-α), both in vivo and in vitro, exhibiting good anti-inflammatory activity.

#### 2.2.3. Anticancer Effect

Polyphenols have good protective effects against some types of cancer. They can inhibit tumor proliferation and have toxic effects on cells, inducing apoptosis. Lee et al. [49] reported that resveratrol can affect the course of cancer and other related diseases through its inhibitory effect on cell proliferation, its apoptotic effect, and its good antioxidant properties. Their report also indicated that quercetin has been widely used in the prevention and treatment of esophageal cancer. The anticancer activity of polyphenolic compounds has also been confirmed in several other studies. For example, silymarin has been observed to induce apoptosis in liver cancer cells and has shown good preventive and therapeutic effects against liver diseases, and epigallocatechin gallate (EGCG) and curcumin have good anticancer effects on breast cancer [51,52].

#### 2.2.4. Antimicrobial Effect

Polyphenols have good antibacterial effects on a variety of microorganisms. This is especially the case for flavonoids, which have significantly higher antibacterial activity than some other polyphenolic compounds. Some reports have shown that polyphenolic compounds can synergize with antibiotics and exhibit excellent antibacterial properties. Liu et al. [55] reported that chitosan-film-loaded curcumin showed good antibacterial activity against *Staphylococcus aureus* and *Rhizopus solani*. In addition, the antibacterial activities of tea polyphenols, silymarin, and rutin have been widely reported and confirmed [41,46,56].

#### 2.2.5. Pro-Oxidant Effect

Polyphenolic compounds have excellent antioxidant effects. However, some polyphenolic compounds can cause DNA damage at high doses, even to the point of causing apoptosis [75]. Canedo-Santos et al. [57] showed that the pro-oxidant properties of resveratrol accelerated the aging process and its pro-oxidant activity shortened the chronological life span of brewer’s yeast. In contrast, gallic acid, while inhibiting lipid oxidation and protein carbonyl formation, tends to promote the oxidative loss of thiol and amine groups, thereby affecting the structural properties and biological activity of proteins [58]. The addition of high doses of soybean into the feed of meat pigs promoted oxidative changes in pork fat, liver, and plasma, and their addition for 64 days increased superoxide dismutase activity and total antioxidant capacity, showing strong pro-oxidant activity [59]. In view of this, the dosage of polyphenolic compounds used in applications is an important point of attention.

#### 2.2.6. Antidiabetic Effect

A diet rich in polyphenols may reduce the risk of diabetes. Some studies have shown that polyphenols can regulate the insulin pathway and enhance insulin sensitivity in the periphery of tissues [76]. Many polyphenolic compounds demonstrate strong inhibition of alpha amylase and alpha glucosidase, which regulate the intestinal absorption of glucose and maintain blood sugar balance [77]. Some foods, such as tea, are rich in a variety of polyphenolic compounds including catechins, which have excellent antioxidant and antidiabetic activities. The therapeutic potential of quercetin as an antidiabetic bioactive substance was reported in detail in a review by Eid et al. [64]. A comprehensive and systematic summary and description of the mechanism of action, the targets, and the effects of quercetin were reported, showing that quercetin has, in vitro and in vivo, been found to have good preventive and therapeutic potential against diabetes [64]. Thus, polyphenolic compounds have great potential for application in the prevention and control of diabetogenesis.

#### 2.2.7. Antihypertensive Effect

Some polyphenolic compounds, such as cocoa that is rich in flavanol compounds including catechins and proanthocyanidins, can effectively improve endothelial function, effectively reduce the oxidative sensitivity of low-density lipoproteins, and increase vasodilation. This effect has been recognized by the European Food Safety Authority [78]. A report by Huang et al. [68] described in detail the beneficial effects of flavonoids, flavanols, anthocyanins, phenolic acids, tannins, resveratrol, and other polyphenolic compounds on vasodilation and other aspects of blood pressure control. In another study, researchers evaluated the vasodilatory effects of curcumin, amlodipine, and curcumin and amlodipine in combination on isolated rat aortic rings, and found that hypertensive patients taking amlodipine could consume curcumin or turmeric for food or other medical purposes without inhibiting the antihypertensive effects of amlodipine [65]. This provides a valuable basis for the use of curcumin and other polyphenolic compounds with antihypertensive effects as food ingredients for the prevention and treatment of hypertension.

#### 2.2.8. Antiobesity Effect

Polyphenols can affect obesity through various effects, such as inhibiting adipocyte proliferation, stimulating adipocyte apoptosis, promoting lipolysis, and fat oxidation. Green tea is a beverage with a variety of health benefits and is a rich source of gallocatechin gallate and catechin, as well as a variety of polyphenolic compounds such as catechins, and can play an important role in obesity control [79]. Evidence suggests that soybean is able to control obesity by inhibiting the activity of pancreatic lipase and pancreatic protein lipase, thereby inhibiting adipocyte differentiation, and by activating hormone-sensitive lipase to stimulate lipolysis [69]. Similarly, in a study by Ting et al. [70], quercetin supplementation was found to have a significant inhibitory effect on the accumulation of adipose tissue in obese rats, with potential anti-obesity effects.

#### 2.2.9. Antiatherosclerotic Effect

A variety of plant polyphenolic compounds can protect the cardiovascular system through different mechanisms, such as increasing high-density lipoprotein, decreasing low-density lipoprotein, and preventing low-density lipoprotein oxidation [80]. For example, ellagic acid and resveratrol can play an anti-atherosclerotic role by improving endothelial barrier function [72,74,81]. In addition, according to the research of Tanaka et al. [73], EGCG has also been found to exhibit certain anti-atherosclerotic effects, and this mechanism of action was elaborated and demonstrated in detail in the report. Polyphenolic compounds have shown good effects in the prevention and treatment of atherosclerosis, and have attracted attention in the development and application of functional foods.

## 3. Common Bio-Based Polymer Nano-Delivery of Polyphenolic Compounds

**Figure 3:**
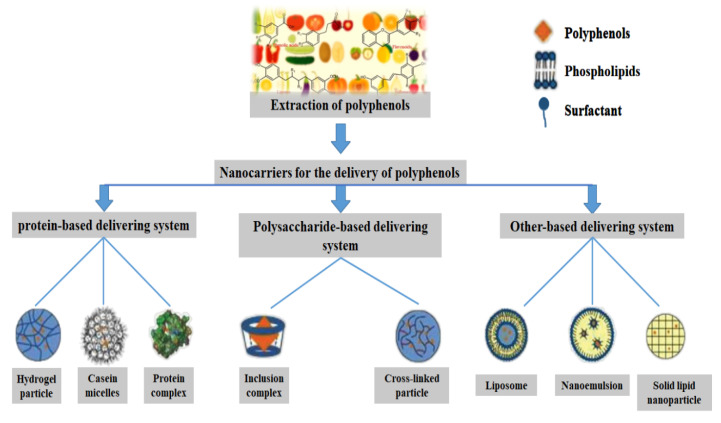
Different types of bio-based nanocarriers for delivery of polyphenols.

Biopolymers have many excellent properties, such as being biodegradable and bio-compatible, and are good choices as delivery carriers for polyphenolic compounds. Among several types of biopolymers, the most frequent and extensive studies have been of protein-based, polysaccharide-based, and lipid-based carriers (Table 3). To enhance the trend of including polyphenolic compounds in nutraceuticals, some limitations in their application have been weakened or overcome. The effective design and preparation of nanocarriers using a variety of biomolecules such as proteins, polysaccharides, and lipids for the encapsulation, protection, and delivery of polyphenolic compounds has been widely studied (Figure 3) [1].

It is encouraging that some commercialized functionalized products containing polyphenols have been introduced onto the market (Table 4), which undoubtedly provides a point of reference for the further exploration and development of new polyphenol health nutrition products [98]. WackerChemie AG has developed CAVACURCUMIN@ for enhanced absorption in combination with γ-cyclodextrin [15]. New formulations containing curcumin nanoparticles, such as Theracurcumin^®^, a brand name of curcumin nanoparticles, have also shown improved pharmacokinetic properties in clinical trials with human volunteers [16]. Preparation of polyphenol nanocomposites using nanotechnology has significantly improved the physicochemical properties of the compounds and provided unique characteristics in terms of size, surface, drug delivery and targeting potential. Polyphenol-supported biological nanocomposites have broad application prospects for health care products, functional food and cosmetics. This phenomenon provides continuous support for the development of food-grade bio-based nanocarriers.

Different types of bio-based nanocarriers can be used as embedding agents and protective barriers for plant polyphenols, which can be divided into nanoparticles, nanomicelles, nanogels, nanoemulsions, and liposomes. A range of nanocarriers, including zein, soy protein, albumin, starch, cellulose, and lipids have been shown to efficiently deliver polyphenolic compounds and improve their bioavailability [1]. Different packaging materials have different effects on the physical and chemical properties of the carrier. Some studies have found that protein and chitosan are more popular as materials for carrier preparation, and extensive studies have reported their favorable effects on increasing intestinal absorption and the bioavailability of plant polyphenols [14]. The encapsulation mechanisms of polyphenols by nanocarriers are diverse, including ionic gel, eutectic, condensation, encapsulation, emulsification, freeze-drying, and yeast encapsulation. These encapsulation mechanisms can improve the water dispersion, gastrointestinal environmental stability, targeting, and sustained release of polyphenols [1]. For example, between curcumin and zein there exists hydrogen bonding, along with electrostatic interaction, and hydrophobicity, which are the main driving forces of nanoencapsulation [82].

Proteins are commonly used for the encapsulation and delivery of polyphenolic compounds, due to their many excellent properties such as high nutritional value, low toxicity, biocompatibility, and biodegradability. They are designed in various forms (nanoparticles, thin films, hydrogel fibers, etc.) and can also be modified or compounded to improve their surface functionality, thus achieving increased bioavailability of polyphenolic compounds [99]. Lozano-Pérez et al. [100] conducted encapsulation, adsorption, and release studies on quercetin using nanoparticles prepared from silk proteins, and the encapsulated quercetin maintained good free radical scavenging ability and was able to achieve a slow-release effect in simulated intestinal fluids. Similarly, whey protein nanoparticles are a suitable carrier for the encapsulation, delivery, and slow gastrointestinal release of polyphenolic compounds such as curcumin, and the system can maintain good stability at pH 7, which provides a good theoretical basis for its application in functional beverages [101]. Other proteins, including bovine serum albumin [102] and zein [99], have often been used as materials for the preparation of nanocarriers to encapsulate polyphenol compounds and improve their bioavailability.

Polysaccharides have likewise received much attention as raw materials for the preparation of bio-based nanoparticles. Various technological approaches allow the modification of polysaccharide-based nanomaterials suitable for the encapsulation and delivery of bioactive molecules in various fields, especially for the protection of polyphenolic compounds [103,104]. Geetha et al. [105] designed and prepared curcumin-loaded starch nanoparticles from cassava starch, and the successful loading of curcumin was confirmed by fluorescence spectroscopy and Fourier infrared transform. Chitosan, a natural polysaccharide with good inter-solubility and biodegradability, has been widely studied for the delivery of polyphenolic compounds. Chitosan nanoparticles can effectively achieve the slow release, controlled release, and improved bioavailability of polyphenolic compounds such as tea polyphenols [106].

As well as proteins and polysaccharides, lipids are another class of bio-based nanocarrier that are commonly used to encapsulate polyphenolic compounds, and are equally biocompatible and biodegradable. Bioactive systems based on solid lipid nanoparticles have been shown to be effective in delivering hydrophobic nutraceuticals. For example, Qin et al. [95] used solid lipid nanoparticles to encapsulate and deliver resveratrol, and found that resveratrol-loaded solid lipid nanoparticles were effective in enhancing antioxidant defense and conferring anti-fatigue capacity after extensive exercise in mice, which provides a valuable reference for resveratrol delivery systems within the field of novel anti-fatigue sports nutrition. Similarly, the bioavailability of other polyphenolic compounds such as quercetin was enhanced to varying degrees by the solid lipid nanocarriers [107]. In addition, nanoemulsions and liposomes are also effective delivery systems for lipophilic polyphenolic compounds, and provide excellent support for the enhancement of plant polyphenol bioavailability. In the study by Li et al. [96], nanoemulsions were extensively studied for the encapsulation, protection, and delivery of lipophilic functional components including polyphenols, pigments, and flavors. Polyphenolic compounds delivered via nanoemulsions have the ability to improve the solubility of hydrophobic compounds, providing better kinetic and biological effects. This gives a key reference for the use of nanoemulsions in the application of plant polyphenols as nutritional formulations in the food industry. Liposomes have also recently been studied for the delivery of different functional compounds in food systems; their biodegradability, non-toxicity, small size, and unique amphiphilic nature were found to confer excellent delivery [97]. Nowadays, the application of lipid delivery systems for the bioavailability enhancement of polyphenolic compounds has been extensively studied, laying the theoretical foundation for the application of polyphenolic compounds in nutraceuticals.

In addition, some protein-based nanoparticles encapsulated with polysaccharides can better improve the stability of delivery systems, such as the preparation of polysaccharide–protein composite nanoparticles using pectin [108], carrageenan [109], chitosan [110], etc., in complex with zein. This can improve the limitation of single zein nanoparticles that tend to aggregate, and then enhance the delivery capacity and protection effect of composite particles to polyphenol compounds. Dai et al. [111] prepared zein-rhamnoid composite nanoparticles by combining zein with rhamnolipid. The study found that the composite nanoparticles had a good encapsulation and protection effect on curcumin, providing an alternative food-grade nano-carrier for the delivery of hydrophobic health care products in functional food and beverages.

In conclusion, biomolecule-based nanoparticles have many advantages, such as environmental friendliness, biocompatibility, and abundant obtainability, which make them excellent materials for the application of polyphenol compound delivery carriers in dietary foods. The continuous development and gradual maturation of nanotechnology has greatly reduced the limitations associated with the low bioavailability of polyphenolic compounds, and the design and preparation of food-grade polyphenol-bio-based nanocomposite systems have become important areas in the development of functional foods.

## 4. Conclusions

Polyphenolic compounds are receiving increased attention due to their wide distribution in plants and their good biological activities. As the application of nanotechnology in the delivery of bioactive ingredients has gradually matured, the development and design of food-grade nanocarriers has gradually advanced, opening up broad application prospects for polyphenols as functional food ingredients. With the increasing pursuit of food diversification and functionalization, some functional foods and special dietary foods designed with plant polyphenols as their main active ingredients have received relevant safety certifications and are widely appreciated. However, certain other polyphenolic compounds, which also have excellent biological activities and functional properties that have been confirmed by researchers, have not yet been approved for food addition. This continues to pose limitations for the development of functional foods. This paper has reviewed the types of polyphenolic compounds and health benefits, including antioxidant, anticancer, antibacterial, antiviral, antiatherosclerotic, and anti-obesity effects. It has discussed the promising development and application of plant polyphenols encapsulated, protected, and delivered through nanocarriers as food ingredients in sustainable and healthy functional foods. This paper also looks forward to the broad prospects of plant polyphenols as food ingredients as part of the development and application of healthy, environmentally friendly functional foods, hoping to provide new insights and research ideas for the development of polyphenolic compounds in food applications. In addition, the potential nanotoxicity of polyphenol nanocomposites is also a concern. The development of commercial polyphenol nanomaterials must be subject to adequate safety assessment. Regulators should take steps to establish standardized safety assessment guidelines so that their entry into the market can be appropriately monitored.

## Figures and Tables

**Table 1:** Chemical structure, physiological activity, and food sources of flavonoid compounds.

Flavonoids	Chemical Structure	Biological and Pharmacological Activity	Refs
Quercetin	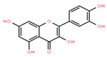	Promote antioxidant, anti-inflammatory, immunoprotective effects, anticarcinogenic, antidiabetic activities	[19]
Rutin	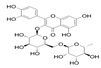	Anti-inflammation, antioxidation, antiallergy, antivirus	[20]
Baicalin	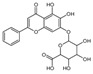	Antibacterial, diuretic, anti-inflammatory, cholesterol-lowering, antithrombotic, asthma relief, fire relief and detoxification, hemostasis	[21]
Myricetin	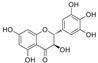	Anti-inflammatory, antitumor, antimutagenic, caries prevention, antioxidant properties, elimination of free radicals in the body	[22]
Fisetin	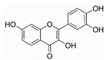	Anti-inflammatory, antioxidant, anticoagulant, antithrombotic, antispasmodic, treatment of diabetic kidney injury	[23]
Liquiritigenin	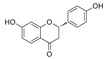	Antispasmodic, anti-ulcer, antibacterial, hepatocyte monoamine oxidase inhibitor	[24]
Apigenin	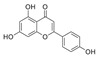	Anticancer, antiviral drug, anti-inflammatory, antioxidant, sedative, tranquilizer, antihypertensive	[25]
Luteolin	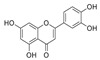	Anti-inflammatory, antitumor, antiallergy, treatment of demyelinating disease, anti-inflammatory chemistry, uric acid lowering	[26]
Morin	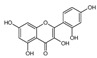	Anti-inflammatory, immunomodulatory effect, antitumor effect, antioxidant effect	[27]
Daidzein	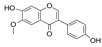	Antioxidant, estrogenic effects	[28]
Puerarin	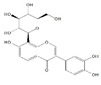	Anti-inflammatory, anticancer, cardiovascular disease prevention	[29]
Genistein	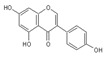	Antioxidant, estrogenic, and antihormonal properties, anticancer activity	[30]
Biochanin A	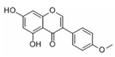	Estrogen-like effect, can inhibit the rise of cholesterol, also has anti-fungal and antitumor effects	[31]
Delphinidin	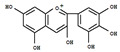	Antioxidants, which can protect the body from damage caused by harmful substances including free radicals	[32]
Cyanidin-3-O-glucoside	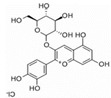	Antioxidant, antitumor, neuroprotective, restores transient vision loss	[33]
Tangeretin	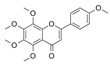	Antifungal effect, antitumor, inhibition of smooth muscle contraction	[34]
Hesperidin	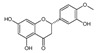	Lower blood pressure, anti-allergy, lower bone density, cholesterol, antibacterial, anti-inflammatory, antihepatitis, antitumor	[35]
Catechin	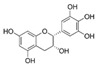	Antioxidant, age-delaying, obesity control, antibacterial	[36]
Isobavachalcone	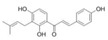	Antibacterial, anticancer	[37]

**Table 2:** Biological activity and health benefits of polyphenols.

Beneficial Effect	Polyphenols Examples	References
Antioxidant	Curcumin	[38]
	Catechin	[39]
	Epigallocatechin gallate (EGCG)	[40]
	Silymarin	[41]
	Apigenin	[42]
Anti-inflammatory	Curcumin	[43]
	Resveratrol	[44]
	Baicalin	[45]
	Rutin	[46]
	Hesperetin	[47]
	Silybin	[48]
Anticancer	Resveratrol	[49]
	Quercetin	[50]
	Curcumin	[51]
	EGCG	[52]
	Hypericin	[53]
	Silymarin	[54]
Antimicrobial	Curcumin	[55]
	Silymarin	[41]
	Tea polyphenol	[56]
	Rutin	[46]
Pro-oxidant	Resveratrol	[57]
	Gallic acid	[58]
	Daidzein	[59]
	Curcumin	[60]
Antidiabetic	Catechin	[61]
	EGCG	[62]
	Curcumin	[63]
	Quercetin	[64]
Antihypertensive	Curcumin	[65]
	Tea polyphenol	[66]
	Procyanidin	[67]
	Resveratrol	[68]
Antiobesity	Daidzein	[69]
	Curcumin	[63]
	Quercetin	[70]
	Catechin	[71]
Antiatherosclerotic	Curcumin	[63]
	Ellagic acid	[72]
	EGCG	[73]
	Resveratrol	[74]

**Table 3:** Common bio-based nano-delivery systems for polyphenols.

Common Bio-Based Nano-Delivery Systems	Types of Carriers	Materials	Major Outcomes	Refs
Protein-based	Nanoparticles Nanogels Nanofilms Nanofibers Nanoemulsion	Zein	Renewable resources with performance and efficiency advantages	[82]
		Soy protein	Various food active ingredients with high nutritional value, functional activity and health effects	[83]
		Rice protein	High value-added protein complexes	[84]
		Ferritin	Natural iron storage protein with a hollow shell for encapsulation and delivery of bioactive nutrients	[85]
		Albumin	Safe and well-tolerated in humans	[86]
		Gliadin	Natural and sustainable resources; environmentally friendly and safe manufacturing process; good biocompatibility	[87]
		Casein	With both hydrophilic and hydrophobic properties	[88]
		Whey Protein	Binding ability to hydrophobic active substances, gelation and emulsification properties	[89]
Polysaccharide-based	Nanoparticles Nanogels Nanofilms Nanofibers Nanoemulsion	Starch	Wide range of raw material sources; non-toxic, biocompatible, ideal material choice for nano-delivery carriers	[90]
		Cellulose	Natural fiber extraction, high crystallinity and high Young’s modulus	[91]
		Lignin	Amphiphilic nanoparticles with multiple interactions with hydrophobic and hydrophilic polyphenols	[92]
		Marine Polysaccharides	Versatility and good biocompatibility as a wall material for colon-targeted delivery of polyphenols for disease intervention	[93]
		Glycogen	Molecular weight is above the renal threshold and is restricted to renal clearance in the blood stream without biodegradation	[94]
Lipid-based	Solid Lipid Nanoparticles Nanoemulsion Liposomes	Lipid compounds	Enhances the resistance of active molecules to environmental, enzymatic, and chemical static stress; improves intestinal solubility; provides a larger surface-to-mass ratio; increases intestinal absorption	[95,96,97]

**Table 4:** Commercialized nano-polyphenols [98]. Reprinted from Rambaran T.F. (2022), copyright (2022), with permission from Elsevier.

Nano-Formulation Trade Name	Form	Main Functional Component	Country Manufactured
CurcuminRich Theracurmin^®^	Capsule	Optimized curcumin	Canada
CAVACURCUMIN^@^	Capsule	Curcumin	Germany
Theracurcumin^®^	Capsule	Curcumin	USA
Turmeric ultra-Nano Curcumin with Piperine	Capsule	Nano curcumin	Jordan
Nano Curcumin	Capsule	Nano curcumin	India
Nano Curcumin Plus	Capsule	Nano curcumin	USA
Nanocurcumin Double Plus	Capsule	Nano curcumin extract	Vietnam
Nano-curcumin	Liquid (Drink)	Curcuminoids	Sweden
Nanocumin Super Food	Liquid (Drink)	Turmeric powder extract	South Korea
Healing Cell Gold Nano CurcuminSerum	Liquid (Serum)	Curcumin extract	Singapore
Nano Resveratrol Facial Serum	Oil	Nano resveratrol	Brazil
CumarGOLD Gel-Nano Curcumin Skincare	Gel	Nano curcumin	Vietnam
Nano Food	Liquid (Oral supplement)	Polyphenol-rich extract	Indonesia
Nano Resveratrol (Nano Red Wine)	Liquid (wine)	Nano resveratrol, grape seed extract	Japan
Nanoemulsified Milk Thistle	Liquid (Oral supplement)	Milk thistle seed extract	USA
Nano Food Kids	Liquid (Oral supplement)	Polyphenol-rich extract	Indonesia
Nanoceuticals™ Slim Shake Chocolate	Liquid (drink)	Polyphenol-rich extract	USA
Alqunus Nano Curcumin	Liquid (Oral supplement)	Turmeric	India

## Data Availability

Not applicable.
